# High Blood Pressure Among Emergency Department Patients: Effects of Educational Intervention

**DOI:** 10.7759/cureus.89240

**Published:** 2025-08-02

**Authors:** Catherine A Marco, Grace Wang, Gavin Schaefer-Hood, Matthew Turner, Matthew Egner, Alexander Haynos, Caroline Soderman

**Affiliations:** 1 Emergency Medicine, Penn State Health Milton S. Hershey Medical Center, Hershey, USA; 2 Emergency Medicine, Penn State College of Medicine, Hershey, USA

**Keywords:** education, emergency, high blood pressure, hypertension, public health

## Abstract

Introduction

Hypertension is a significant global health issue. It is estimated that approximately half of adults in the United States have hypertension (approximately 119 million adults). Although emergent treatment is not indicated for asymptomatic hypertension, patients presenting to the emergency department (ED) with hypertension may represent an opportunity for patient education and improved health outcomes. This study was undertaken to identify healthcare outcomes associated with an educational intervention for patients presenting to the ED with hypertension.

Methods

In this prospective interventional study, subjects included consenting adult Emergency Department (ED) patients with hypertension (defined by the International Society of Hypertension as systolic blood pressure (BP) over 140 mm Hg and/or diastolic BP over 90 mm Hg), who were discharged home. Baseline assessment of medication adherence, exercise, diet, tobacco, and alcohol use was performed. An educational intervention was provided using the American Heart Association: Blood Pressure Fact Sheets. Subjects were contacted at two and four weeks following the ED visit to assess BP, medication adherence, exercise, diet, and tobacco and alcohol use.

Results

Among 151 participants, data were available at two weeks for 89 subjects (59% follow-up rate). Following the educational intervention, participants had lower systolic BP at two weeks (baseline mean: 163 mm Hg (95% CI: 159-166), two-week mean: 130 mm Hg (95% CI: 126-135); p < 0.001) and diastolic BP at two weeks (baseline mean: 93 (95% CI: 91-95); two-week mean: 77 mmHg (95% CI: 75-80); p < 0.001). Participants were more likely to take antihypertensive medication daily at two weeks (baseline: 52%; two-week mean: 69%; p = 0.0271). There was no significant difference in self-reported healthy diet (baseline: 21%; two-week mean: 26%; p = 0.1). There was no significant difference in self-reported exercise (baseline: 30%; two-week mean: 26%; p = 0.8). There were no differences in smoking or alcohol use at two weeks. Similar results were found at four weeks. Data were available at four weeks post intervention for 88 subjects (58.2% follow-up rate). At four weeks, participants had a lower systolic BP (baseline: 163 mmHg (95% CI: 159-166); four-week mean: 132; p < 0.001) and lower diastolic BP (baseline: 93 mmHg (95% CI: 91-95); four-week mean: 78; p < 0.001). A significant difference in self-reported exercise emerged at four weeks, with a lower percentage reporting no exercise (baseline reporting no exercise: 30%; four-week mean: 18%, p = 0.0348). There were no significant differences in reported healthy diet adherence (baseline: 21%; four-week mean: 24%; p = 0.6705), smoking (baseline: 11.92%; four-week mean: 5.68%; p = 0.1147), or alcohol use (baseline: 34%; four-week mean: 28%; p = 0.3361).

Conclusions

An educational intervention in the ED was associated with lower systolic and diastolic BP at two and four weeks post ED visit, improved medication adherence at two and four weeks, and self-reported exercise at four weeks. There were no differences in reported healthy diet adherence, exercise, smoking, or alcohol use.

## Introduction

Hypertension is a common disease in the United States. Approximately 31-50% of adults experience some form of hypertension; among these, anywhere from 41-50% do not have their blood pressure (BP) under adequate control [1-4]. Hypertension is associated with increased risk of stroke, heart disease, kidney disease, and other conditions.

Asymptomatic hypertension is common in the Emergency Department (ED); a recent study found that 49% of patients discharged from the ED had elevated BP readings and that 73% had no prior diagnosis of hypertension [5]. Previous studies have shown that patients without a known diagnosis of hypertension presenting with elevated BP to the ED (defined as 140/90) are likely to have significantly elevated pressures at home as well, indicating that these elevated pressures cannot be dismissed as due to pain or anxiety in the ED setting [6]. A recent 2024 retrospective survey study of 167 patients found that BP ≥160/100 mmHg at an ED visit can reveal undiagnosed hypertension in one-third of patients [7].

The majority of cases of hypertension that present to the ED are not emergent and do not require immediate intervention. The American College of Emergency Physicians (ACEP) has stated in its Clinical Policy on Asymptomatic High Blood Pressure, “in patients with asymptomatic markedly elevated BP, routine ED medical intervention is not recommended” [8]. The ACEP has recommended that ED physicians refer patients with evidence of hypertension to a primary care provider (PCP). Unfortunately, this policy is inconsistently followed at best [9]. Less than 30% of patients presenting with mild-to-severe hypertension are given referrals for follow-up care, and many patients have difficulty establishing care with a PCP even with a formal referral [10].

Several studies have demonstrated favorable effects of patient education regarding hypertension in the outpatient setting [11-16], and these may be generalizable to the ED setting. These educational interventions play a key role in both increasing patient awareness about their condition, as well as enhancing medication adherence. As many patients visit the emergency department with complaints related to high BP, such as chest pain and shortness of breath, providers are given the opportunity to inform them of the potential dangers and long-term complications related to their hypertension. By providing information, patients are more likely to implement this new understanding into their daily lives, improving their short-term and long-term health outcomes [17]. The ED environment is an opportunity to provide educational healthcare resources, which can benefit a large proportion of the population [18].

This study was undertaken to identify healthcare outcomes, including the primary outcome of BP, as well as secondary outcomes of primary care follow-up, medication adherence, tobacco use, nutrition, and exercise, associated with an educational intervention for patients presenting to the ED with hypertension.

## Materials and methods

This prospective interventional design study was approved by the Penn State University Institutional Review Board. Eligible subjects included adult ED patients, age 18 and over, who had elevated BP (defined by the International Society of Hypertension as systolic BP over 140 mmHg and/or diastolic BP over 90 mmHg) for any single BP reading during their ED stay, in the Milton S. Hershey Medical Center, and were discharged home. Subjects were enrolled from May 2024 through August 2024. Exclusion criteria included patients under age 18, prisoners, patients in distress, patients unable to consent, cognitively impaired adults, and non-English speaking adults.

Trained research assistants identified consecutive ED patients with asymptomatic hypertension as a convenience sample when a research assistant was available. Research assistants were trained in how to administer the tool (American Heart Association: Blood Pressure Fact Sheets) and educate the patients. An educational intervention was then performed, which included a handout developed by the American Heart Association and a discussion of actions to take [19]. This educational tool was utilized due to educational content and readability. Consent was obtained from the American Heart Association to use this educational handout for this research project. Subjects were contacted by telephone at two weeks and four weeks following the ED visit to collect information regarding self-reported outcomes, including BP, lifestyle, and medication compliance with antihypertensive therapy. No additional contact with participants was performed following the initial ED visit.

Baseline information regarding the patient’s medical history, lifestyle factors, and medication adherence using the five‐item Medication Adherence Rating Scale (MARS-5) was collected. The MARS-5 has been validated as a tool to measure medication adherence [20]. Outcome measures include data regarding BP, medication adherence, exercise, diet, and alcohol and tobacco use at two weeks and four weeks following the ED visit.

Data analysis

A power analysis demonstrated that a sample size of 14 would achieve 91% power to detect a mean of paired differences of -2 with an estimated standard deviation of paired differences of 2 and with a significance level (alpha) of 0.05 using a two-sided paired Wilcoxon signed-rank test assuming that the actual distribution of paired differences is normal. Descriptive statistics were generated, including means, medians, standard deviations, and confidence intervals for continuous variables; frequency tables and 95% confidence Intervals were calculated for categorical variables. Subgroup analyses were performed, and t-tests were performed for continuous variables and Pearson’s chi-square tests for categorical variables. Missing data were omitted from analysis. The statistical analysis was performed using SAS software (version 9.4; SAS Institute Inc., Cary, NC).

## Results

Among 151 participants, the mean age was 61 (range: 20-89), approximately half were female (n = 76; 50%), and the majority were White participants (n = 125; 83%) (Table 1). Most participants (n = 108; 72%) reported that they had been previously prescribed medications to treat high BP, and 52% of participants (n = 78) reported currently taking daily hypertension medications. Subjects had elevated BP with mean systolic and diastolic BP of 163 mmHg (SD = 20.8) and 93 mmHg (SD = 15.3), respectively. Regarding lifestyle factors, 30% (n = 45) reported no exercise per week, while 21% (n = 32) reported a healthy diet, 12% (n = 18) smoked tobacco products, and 33% (n = 52) drank alcohol.

**Table 1:** Baseline demographics of the 151 ED patients with high blood pressure HTN: hypertension

Parameters	Total (N=151) n (%)
Age (years)	61 (range: 20-89)
Gender	
Female	76 (50.3%)
Male	75 (49.7%)
Ethnicity	
White	125 (82.8%)
Black	12 (8.0%)
Asian	4 (2.7%)
Other	10 (6.6%)
Prescribed hypertension medication	108 (72.0%)
Currently taking medication for HTN	
Daily	78 (52.0%)
Sometimes misses doses	14 (9.3%)
No	58 (38.7%)
Type of diet	
Healthy	32 (21.2%)
Moderate	90 (59.6%)
Unhealthy	29 (19.2%)
Frequency of exercise per week	
5-7 times	49 (32.5%)
3-4 times	24 (15.9%)
1-2 times	33 (21.9%)
None	45 (29.8%)
Currently smoker	18 (11.9%)
Currently drinker	52 (34.4%)

Data were available at two weeks post intervention for 89 subjects (59% follow-up rate). At two weeks, participants reported lower systolic BP (baseline: 163 mmHg; two-week mean: 130 mmHg; p < 0.001) and lower diastolic BP at two weeks (baseline: 93 mmHg; two-week mean: 77 mmHg; p < 0.001) (Table 2). Approximately 72% of participants were prescribed antihypertensive medications at baseline, with no significant change at two weeks (baseline: 72%; two-week mean: 81%; p = 0.1230). Participants were more likely to be taking antihypertensive medication daily at two weeks (baseline: 52%; two-week mean: 69%; p = 0.027) (Table 3). No significant difference in self-reported exercise level occurred (baseline reporting no exercise: 30%; two-week mean: 26%; p = 0.8). Similarly, no differences were seen at two weeks for self-reported healthy diet (baseline: 21%; two-week mean: 26%; p = 0.1), smoking (baseline: 12%; two-week mean: 9%; p = 0.4803) or alcohol use (baseline: 34%; two-week mean: 31%; p = 0.6366).

**Table 2:** Mean systolic and diastolic blood pressures of participants at baseline and at two- and four-week follow-ups *p < 0.05 considered significant

Baseline			2-week follow-up				4-week follow-up			
Blood Pressure (mm Hg)	M (SD)	95% CI	M (SD)	95% CI	Paired T-test statistic	p value	M (SD)	95% CI	Paired T-test statistic	p value
Systolic	163 (21)	(159-166)	130 (18)	(126-135)	10.21	<0.0001	132 (17)	(128-136)	10.59	<0.0001
Diastolic	93 (15)	(91-95)	77 (11)	(75-80)	9.06	<0.0001	78 (13)	(75-81)	5.88	<0.0001

Data were available at four weeks post intervention for 88 subjects (58.2% follow-up rate). At four weeks, participants had a lower systolic BP (baseline: 163 mmHg, four-week mean: 132 mmHg; p < 0.001) and lower diastolic BP (baseline: 93 mmHg; four-week mean: 78 mmHg; p < 0.001) (Table 2). The proportion of participants prescribed antihypertensives at four weeks was not significantly different (baseline: 72%; four-week mean: 73%; p = 0.9037). Participants were more likely to be taking daily antihypertensive medications also at four weeks (baseline: 52%; four-week mean: 75%; p = 0.0037) (Table 3). A significant difference in self-reported exercise was demonstrated at four weeks, with a lower percentage reporting no exercise (baseline reporting no exercise mean: 30%; four-week mean: 18%; p = 0.0348). No differences were identified in reported healthy diet adherence (baseline: 21%; four-week mean: 24%; p = 0.6705), smoking (baseline: 12%; four-week mean: 6%; p = 0.1147), or alcohol use (baseline: 34%; four-week mean: 28%; p = 0.3361) (Table 4).

**Table 3:** Medication adherence to daily antihypertensive medication *p < 0.05 is considered significant.

Variables	Baseline		2 weeks				4 weeks			
	N (%)	95% CI	N (%)	95% CI	Chi-square statistic	p value	N (%) (95% CI)	95% CI	Chi-square statistic	p value
Have you been prescribed medication to treat high blood pressure?	108 (72)	65-79	64 (81)	63-82	2.38	0.12	64 (73)	63-82	0.01	0.90
Are you currently taking your medication daily to treat high blood pressure?	78 (52)	44-60	61 (69)	59-71	7.22	0.03*	57 (75)	65-85	11.19	0.003*

**Table 4:** Lifestyle factors for participants baseline and at two and four weeks following educational intervention *p < 0.05 is considered significant.

Variables	Baseline		Two Weeks				Four Weeks			
	N (%)	95% CI	N (%)	95% CI	Chi-square statistic	p value	N (%)	95% CI	Chi-square statistic	p value
Diet (Reporting “healthy” diet)	32 (21)	15-28	23 (26)	17-35	4.60	0.1002	21 (24)	15-33	0.80	0.6705
Exercise (Reporting “no exercise” weekly)	45 (30)	22-37	23 (26)	17-35	0.81	0.8466	16 (17)	10-36	8.62	0.0348*
Smoking (Reporting “yes” to smoking)	18 (12)	7-17	8 (9)	3-15	0.50	0.4803	5 (6)	1-11	2.49	0.1147
Alcohol (Reporting “yes” to consuming alcohol)	52 (34)	27-42	28 (31)	22-41	0.22	0.6366	25 (28)	19-38	0.93	0.3361

Responses to the MARS-5 revealed no significant differences at the two- or four-week post-intervention follow-up. No adverse effects were reported by participants.

## Discussion

Hypertension is a growing public health concern and is a modifiable risk factor for cardiovascular disease, which can lead to potentially life-threatening complications if left untreated. A complicating factor in hypertension management and treatment is that many US adults remain unaware that they have high BP. Patients commonly do not experience symptoms of their hypertensive crisis or recognize signs of organ damage. Thus, healthcare encounters provide an opportunity for identification, testing, and management of hypertension, which can avoid future complications if left undiagnosed and unmanaged. The ED is uniquely suited to identify patients suffering from asymptomatic hypertension, as many people utilize the emergency department as primary care, particularly among patients who do not regularly seek care in primary settings. Reports show that lower socioeconomic status correlates with both higher ED utilization and higher rates of hypertension; thus, this setting provides abundant opportunity to identify asymptomatic hypertension [21-23]. These skills include maintaining a healthy diet, regular physical activity, stress management, and medication adherence [24,25]. Studies have shown that simple interventions focusing on lifestyle modifications have been effective in improving self-management behaviors. Interventions that combined education and counseling have been proven to be effective in reducing BP levels [26,27].

This study hypothesized that an educational intervention on patients identified to be hypertensive in the ED setting could positively impact medication adherence and lifestyle factors, leading to sustained improved BP following discharge. The results supported this hypothesis as demonstrated by the significant reduction in systolic and diastolic BP at two weeks and four weeks post-educational intervention compared to baseline. Following educational intervention, which emphasized the importance of consistently taking antihypertensive medications as prescribed, participants were more likely to take antihypertensive medication daily well beyond their ED visit at the two- and four-week follow-ups. The observed reduction in BP, along with the increased medication adherence, aligns with earlier studies that demonstrated the effectiveness of a structured educational program for BP reduction in hypertensive patients [27]. However, the precise impact of education is uncertain as effects may be due to other confounding factors, such as medication compliance, variability in BP readings, or accuracy of self-report. For example, a randomized control trial conducted by Perl et al., consisting of two groups undergoing an educational intervention at two separate time periods, demonstrated improved BP, as well as better adherence to lifestyle modifications [28]. The reasons behind the potential discordance of the self-reported medication compliance and MARS-5 scores are unclear. This may be due to patients' perception of compliance with medication or errors in self-report.

While medication adherence is crucial to lowering BP, medications alone may not sufficiently manage hypertension [29]. Lifestyle modifications such as diet and exercise, while beneficial in lowering BP over time, have been shown to be challenging in habit development. Following educational intervention in the ED, this study found a difference in self-reported exercise at four weeks, but no significant difference compared to baseline in terms of reported diet quality, smoking, or alcohol use at the two- or four-week follow-up. Exercise frequency was also not significant at two weeks. This difference may stem from discrepancies in study design, type of intervention, or, in the timeline of the study, as data were collected at two and four weeks compared to six and 12 months. A decreased locus of control for certain individuals was mentioned as a barrier in the free response during follow-up phone calls. This psychological factor has been shown to play a crucial role in healthy habit development, such as diet and exercise [30].

Our study and intervention failed to show an improvement in lifestyle modifications such as reported diet quality, exercise frequency, and amount of smoking and alcohol use by two weeks. Although lifestyle modifications are important, they can be difficult to implement in such a short period of time and with one-time education. The timeframe of this study may have been a limitation in seeing these improvements. Additionally, people may experience other socioeconomic factors hindering their ability to implement lifestyle changes, such as time constraints, living in a food desert, financial restrictions, and lack of social support. Regular follow-up messages or repeating education at future emergency department visits may result in better outcomes and a potential improvement to this intervention moving forward.

Educational interventions are one way to address the worsening hypertension crisis in the United States. While there are significant constraints to patient education in the ED environment, such as time constraints or the lack of established physician-patient relationships, the unique interface of patients with the health care system in the emergency department offers an opportunity for meaningful intervention and patient education to improve health outcomes related to hypertension. The results of this study, as well as prior publications, support the effectiveness of educational intervention on modifiable behaviors to help reduce BP and increase medication adherence in a short timeframe. However, there are other behaviors that can be modified to further improve patient health outcomes that need to be addressed. Future directions should explore interventional methods to improve diet, exercise, smoking, and alcohol and explore the longevity of educational effects on BP control after discharge. In addition, potential effects of longitudinal follow-up with multiple educational interventions and follow-up data may prove important for long-term BP control.

Limitations

This study was conducted at a single institution, and results may not be applicable in other settings. The convenience sample may have biased results based on research assistant availability. The original BP measurements and survey were conducted by a research assistant in the ED, but the following BP measurements were self-reported by participants over the phone, which could bias results to or away from the null due to recall bias or the Hawthorne effect. Repeat BP measurements may represent a regression to the mean rather than a true decrease in BP. Other limitations include a follow-up rate of 59% at two weeks and 58% at four weeks, which could bias our results to people who might be more self-motivated to be on top of modifiable behaviors. Barriers to establishing primary care (e.g., transportation or financial challenges) may have affected results. Another limitation was that this study was based on participants’ self-reported data for quality of diet, exercise frequency, smoking, and alcohol use, and these data are dependent on the veracity of patient self-reports.

## Conclusions

An educational intervention in the ED was associated with lower systolic and diastolic BP at two and four weeks post ED visit, improved medication adherence at two and four weeks, and self-reported exercise just at four weeks. Participants were more likely to take antihypertensive medication daily at two weeks. There were no differences in reported healthy diet adherence, smoking, or alcohol use at two weeks. Participants were more likely to report exercise at four weeks. An educational intervention in the ED was associated with improved BP and medication compliance.
